# Using Data Mining to Detect Health Care Fraud and Abuse: A Review of Literature

**DOI:** 10.5539/gjhs.v7n1p194

**Published:** 2014-08-31

**Authors:** Hossein Joudaki, Arash Rashidian, Behrouz Minaei-Bidgoli, Mahmood Mahmoodi, Bijan Geraili, Mahdi Nasiri, Mohammad Arab

**Affiliations:** 1 Department of Health Management and Economics, School of Public Health, Tehran University of Medical Sciences, Tehran, Iran; 2 School of Computer Engineering, Iran University of Science and Technology, Tehran, Iran; 3 Department of Epidemiology and Biostatistics, School of Public Health, Tehran University of Medical Sciences, Tehran, Iran; 4 Mazandaran University of Medical Sciences, Mazandaran, Iran

**Keywords:** health care, data mining, KDD, Business Intelligence, insurance claim, fraud

## Abstract

Inappropriate payments by insurance organizations or third party payers occur because of errors, abuse and fraud. The scale of this problem is large enough to make it a priority issue for health systems. Traditional methods of detecting health care fraud and abuse are time-consuming and inefficient. Combining automated methods and statistical knowledge lead to the emergence of a new interdisciplinary branch of science that is named Knowledge Discovery from Databases (KDD). Data mining is a core of the KDD process. Data mining can help third-party payers such as health insurance organizations to extract useful information from thousands of claims and identify a smaller subset of the claims or claimants for further assessment. We reviewed studies that performed data mining techniques for detecting health care fraud and abuse, using supervised and unsupervised data mining approaches. Most available studies have focused on algorithmic data mining without an emphasis on or application to fraud detection efforts in the context of health service provision or health insurance policy. More studies are needed to connect sound and evidence-based diagnosis and treatment approaches toward fraudulent or abusive behaviors. Ultimately, based on available studies, we recommend seven general steps to data mining of health care claims.

## 1. Introduction

### 1.1 Defining Fraud and Abuse

Inappropriate payments by insurance organizations or third party payers occur as a result of error, abuse or fraud. Abuse describes provider’s practices that, either directly or indirectly, result in unnecessary costs to the payer. Abuse includes any practice that is not consistent with the goals of providing patients with services that are medically necessary, meet professionally recognized standards, and are fairly priced (Centers for Medicare and Medicaid Services, 2012).

Health care fraud is an intentional deception used in order to obtain unauthorized benefits (Busch, 2007). Unlike error and abuse, fraudulent behaviors are usually defined as a crime in law. However, there is no global consensus on the definition of fraud and abuse in health care services or health insurance setting. For more details and examples of fraud and abuse, please see Rashidian, Joudaki, and Vian (2012).

It is estimated that about 10 per cent of health care system expenditure is wasted due to fraud and abuse (Gee, Button, Brooks, & Vincke, 2010). Therefore, the scale of health care fraud and abuse is large enough to make it a priority issue for health systems.

### 1.2 Emerging Data Mining for Better Detection of Health Care Fraud and Abuse

In traditional methods of health care fraud and abuse detection, a few auditors handle thousands of paper health care claims. In reality, they have little time for each claim, focusing on certain characteristics of a claim without paying attention to the comprehensive picture of a provider’s behavior (Rashidian et al., 2012). This method is time-consuming and inefficient. It is still the dominant picture in many low-income and middle-income countries (Copeland, Edberg, Panorska, & Wendel, 2013; Aral, Güvenir, Sabuncuoğlu, & Akar, 2012; Ortega, Figueroa, & Ruz, 2006).

Electronic health records and growing use of computerized systems has led to newly emerging opportunities for better detection of fraud and abuse. Innovations in machine learning and artificial intelligence bring attention to automated methods of fraud detection. Combining automated methods and statistical knowledge led to a newly emerging interdisciplinary branch of science that is named Knowledge Discovery from Databases (KDD). Data mining is the core of the KDD process.

Data mining can help third-party payers such as health insurance organizations to extract useful knowledge from thousands of claims and identify a smaller subset of the claims or claimants for further assessment and scrutiny for fraud and abuse (Rashidian et al., 2012). In this way, the data mining approach is part of a more efficient and effective IT-based auditing system.

## 2. Scope and Objectives of Our Study

We reviewed studies that achieved better detection of health care fraud and abuse by using data mining techniques. We aimed to identify different approaches of data mining and applied data mining algorithms for health care fraud detection. Our study does not cover financial fraud, which is not specific to the health care providers. In addition, our study does not cover fraud detection in other fields such as credit card fraud, money laundering, telecommunication fraud, computer intrusion and scientific fraud.

## 3. Related Works

Travaille, Müller, Thornton and Hillegersberg (2011) created an overview on fraud detection within other industries, and how they can be applied within the healthcare industry. They mentioned 14 review studies that have reviewed data mining methods in all fraud detection fields (Travaille, Müller, Thornton, & Hillegersberg, 2011). Also, we found two studies that have reviewed Knowledge Discovery from Databases (KDD) and data mining in health care (Esfandiary, Babavalian, Moghadam, & Tabar, 2013; Yoo et al., 2012). Ultimately, we found three studies that reviewed data mining methods in health care fraud detection (Liu & Vasarhelyi, 2013; Li, Huang, Jin, & Shi, 2008; Furlan & Bajec, 2008).

Our study focused on primary researches that applied data mining methods in health care setting and health insurance. We excluded studies that did not have original data (e.g. Thornton, Mueller, Schoutsen, & van Hillegersberg, 2013; Ogwueleka, 2012; Ormerod, Morley, Ball, Langley, & Spenser, 2003).

## 4. Data Mining (DM), Knowledge Discovery from Databases (KDD) and Business Intelligence (BI)

Nowadays, data mining methods are the core part of the integrated Information Technology (IT) software packages that are sometimes called “Business Intelligence” (BI) (Please see Chee et al. (2009) for a summary of varied BI definitions and approaches to the definition of BI). Usually these IT-based systems have three layers, starting with data warehousing, followed by On Line Analytical Process (OLAP) and ending with data mining methods (that are the most advanced) (Fisher, Lauria, & Chengalur-Smith, 2012; Maimon & Rokach, 2010; Zeng, Xu, Shi, Wang, & Wu, 2006).

In the first layer of analysis, the physician’s claims are compared with pre-computed aggregates along data dimensions (predefined rules) and the system detects certain errors and inconsistency in claims. For example, the price of a drug is defined 10 dollars and the system identifies all of the claims that containthis drug and also break this rule. Reports that are generated by this layer of analysis can help to identify erroneous or incomplete data input, duplicate claims, and services with no medical coverage (Li et al., 2008). Despite of the fact that repeated or frequent errors are susceptible for abuse or fraud, the capability of this analysis layer for detection of fraud and abuse is usually limited (Li et al., 2008).

In the second layer OLAP multi-level is performed (for example presenting the five physicians with the highest rate of prescription of injectable antibiotics compared with the month before). However, providing solutions when the user is unable to describe goals in terms of a specific query is impossible. These two layers of analysis are often unsuccessful in detecting well-documented fraudulent claims and new patterns of fraud and abuse.

The third layer of analysis uses data mining techniques that are more sophisticated compare to the two previous layers. Data mining involves the use of methods that explore the data, develop relevant models and discover previously unknown patterns in the data (Maimon & Rokach, 2010). For example, by using association rules and induction methods one could understand physicians’ prescription behavior (or pattern) and then find which one or group of physicians differ abnormally from the other physicians.

Some researchers have defined data mining as a key part of a broader term of Knowledge Discovery from Databases (KDD) (Maimon & Rokach, 2010; Fayyad, Piatetsky-Shapiro, & Smyth, 1996). Maimon and Rokach (2010) have defined KDD as an organized process of identifying valid, novel, useful, and understandable patterns from large and complex datasets. They have defined Data Mining (DM) as a core of the KDD process, involving the inferring of algorithms that explore the data, develop the model and discover previously unknown patterns (Maimon & Rokach, 2010).

KDD involves several steps, starting from understanding the organization environment, determining obvious objectives, understanding the data, cleaning, preparation and transformation of the data, selecting the appropriate data mining approach, applying data mining algorithms, and evaluation and interpretation of the findings (Rashidian et al., 2012; Maimon & Rokach, 2010). Some researchers have described similar steps as a data mining process (Li et al., 2008). Others have described similar steps for BI (Zeng et al., 2006). Despite of the fact that data warehousing experts, data mining experts, machine learning experts and other experts may view these steps from their own viewpoints or emphasize on some steps as opposed to other steps, the logic and essence of all of these terms is the same. They are all about learning. How a health care organization or insurance organization learns about thousands of claims and makes informed and intelligent decisions. How an organization develop a brain for itself to gather big and different data, analyze the data and respond timely and accurately. We go forward with the data mining as a part of KDD process and KDD as a part of a border term of BI. In our view, data mining is embedded in vertical solutions for KDD, BI and Decision Support Systems (DHS).

## 5. Finding

### 5.1 Classification of Data Mining Methods

There are different classifications of data mining. It depends on the kinds of data being mined, the kinds of knowledge being discovered and the kinds of techniques (algorithms) utilized. The most common and well-accepted categorization that is used by machine learning experts divides data mining methods into ’supervised’ and ’unsupervised’ methods (Phua, Lee, Smith, & Gayler, 2010; Li et al., 2008; Bolton & Hand, 2002). Supervised methods attempt to discover the relationship between input variables (attributes or features) and an output (dependent) variable (or target attribute). Unsupervised learning methods are applied when no prior information of the dependent variable is available for use.

Supervised methods are usually used for classification and prediction objectives including traditional statistical methods such as regression analysis, discriminant analysis, neural networks, Bayesian networks and Support Vector Machine (SVM). Unsupervised methods are usually used for description including association rules extraction such as Apriori algorithm and segmentation methods such as clustering and anomaly detection.

### 5.2 Supervised Data Mining Methods for Detecting Health Care Fraud and Abuse

In the domain of health care fraud and abuse detection, supervised data mining involves methods that use samples of previously known fraudulent and non-fraudulent records. These two groups of records are used to construct models, which allow us to assign new observations to one of the two groups of records. Supervised methods require confidence in the correct categorization of the records. Furthermore, they are useful in detecting previously known patterns of fraud and abuse. Hence, the models should be regularly updated to reflect new types of fraudulent behaviors and changes in the regulations and settings (Rashidian et al., 2012). Examples of the supervised methods that have been applied to health care fraud and abuse detection include decision tree (Shin, Park, Lee, & Jhee, 2012; Liou, Tang, & Chen, 2008; William & Huang, 1997), neural networks (Liou et al., 2008; Ortega et al., 2006; He, Graco, & Yao, 1997), genetic algorithms (He et al., 1999) and Support Vector Machine (SVM) (Kirlidog & Asuk, 2012; Kumar, Ghani, & Mei, 2010) (Please see Table 1).

**Table 1:** Primary studies that used data mining for detecting health care fraud and abuse

Study Topic (Country)	The first author(year)	Data mining approach	Type of detected fraud	Applied data mining technique (s)
Healthcare fraud detection: A survey and a clustering model incorporating Geo-location information (US)	Liu (2013)	Unsupervised	Insurance subscribers’ fraud	Clustering
Application of Bayesian Methods in Detection of Healthcare Fraud (-)	Ekina (2013)	Unsupervised	Conspiracy fraud which involves more than one party	Bayesian co-clustering
Unsupervised labeling of data for supervised learning and its application to medical claims prediction (US)	Ngufor (2013)	Hybrid supervised and unsupervised	Provider fraud (Obstetrics claims)	Unsupervised data labeling and outlier detection, classification and regression
Outlier based predictors for health insurance fraud detection within U.S. Medicaid (US)	Capelleveen (2013)	Unsupervised	Provider fraud (Dental claim data)	Outlier detection
A scoring model to detect abusive billing patterns in health insurance claims (Korea)	Shin (2012)	Supervised	Provider fraud (Outpatient clinics)	Six statistical techniques — correlation analysis, logistic regression and classification tree
A fraud detection approach with data mining in health insurance (Turkey)	Kirlidog (2012)	Supervised	Provider fraud	Support vector machine (SVM)
Applying Business Intelligence Concepts to Medicaid Claim Fraud Detection (US)	Copeland, (2012)	Unsupervised	Provider fraud	Visualization by histogram
A prescription fraud detection model (Turkey)	Aral (2012)	Hybrid supervised and unsupervised	Prescription fraud	Distance based correlation and risked matrices
Unsupervised fraud detection in Medicare Australia (Australia)	Tang (2011)	Unsupervised	Insurance subscribers’ fraud	Clustering, feature selection and outlier detection
Two models to investigate Medicare fraud within unsupervised databases (US)	Musal (2010)	Unsupervised	Provider fraud	Clustering algorithms, regression analysis, and various descriptive statistics
Data mining to predict and prevent errors in health insurance claims processing (US)	Kumar (2010)	Supervised	Error in providers claims	Support vector machine (SVM)
Discovering inappropriate billings with local density based outlier detection method (Australia)	Shan (2009)	Unsupervised	Provider fraud (Optometrists Billing)	Local density based outlier detection
Mining medical specialist billing patterns for health service management (Australia)	Shan (2008)	Unsupervised	Provider fraud (Specialist billing)	Association rules
Detecting hospital fraud and claim abuse through diabetic outpatient services (Taiwan)	Liou (2008)	Supervised	Provider fraud (Diabetic outpatient services)	Logistic regression, neural network, and classification trees
A process-mining framework for the detection of healthcare fraud and abuse (Taiwan)	Yang (2006)	Supervised	Provider fraud (Gynecology services)	Classification based on associations algorithm, feature selection by Markov blanket filter
A medical claim fraud/abuse detection system based on data mining: a case study in Chile (Chile)	Ortega (2006)	Supervised	Provider fraud	Neural network
EFD: A Hybrid Knowledge/Statistical-Based System for the Detection of Fraud (US)	Major (2002)	Hybrid supervised and unsupervised	Provider fraud	Outlier detection and rule extraction
Application of Genetic Algorithms and k-Nearest Neighbour method in real world medical fraud detection problem (Australia)	He (1999)	Unsupervised	Provider fraud (General practitioners)	Genetic algorithm and K-Nearest Neighbor clustering
Evolutionary Hot Spots data mining: architecture for exploring for interesting Discoveries (Australia).	Williams (1999)	Hybrid supervised and unsupervised	Insurance subscribers’ fraud	Clustering and rule induction
Mining the knowledge mine: The Hot Spots methodology for mining large real world databases (Australia)	William (1997)	Hybrid supervised and unsupervised	Insurance subscribers’ fraud	Clustering and C5.0 classification algorithm
Application of neural networks to detection of medical fraud (Australia)	He (1997)	Supervised	Provider fraud (General practitioners)	Neural network

### 5.3 Unsupervised Data Mining Methods for Health Care Abuse and Fraud Detection

When fraudsters become aware of a particular detection method, they will adapt their strategies to avoid detection (Sparrow, 1996). As we noted above, supervised methods are useful in detecting previously known patterns of fraud and abuse. In theory, we can apply unsupervised approaches to identify new types of fraud or abuse.

Unsupervised methods typically assess one claim’s attributes in relation to other claims and determine how they are related to or different from each other. Therefore, it can clear sequence and association rules between records, distinguish anomaly record (s) or group similar records.

Examples of the unsupervised methods that have been applied to health care fraud and abuse are clustering (Liu & Vasarhelyi, 2013; Ekina, Leva, Ruggeri, & Soyer, 2013; Tang, Mendis, Murray, Hu, & Sutinen, 2011; Musal, 2010; C. Lin, C.M Lin, Li, & Kuo, 2008; William & Huang, 1997), outlier detection (Capelleveen, 2013; Tang et al., 2011; Shan, Murray, & Sutinen, 2009) and association rules (Shan, Jeacocke, Murray, & Sutinen, 2008) (Please see Table 1).

### 5.4 Brief Review of Available Studies

We briefly explain some of the studies mentioned in section 5.2 and 5.3. Liou et al. (2008) used supervised methods to review claims submitted to Taiwan’s National Health Insurance for diabetic outpatient services (Liou et al., 2008). They selected nine expense-related variables and compared them in two groups of fraudulent and non-fraudulent claims for building the detection models. The input variables were average drug cost, average diagnosis fee, average amount claimed, average days of drug dispense, average medical expenditure per day, average consultation and treatment fees, average drug cost per day, average dispensing service fees and average drug cost per day. They compared three data mining methods including logistic regressions, neural networks and classification trees for the detection of fraudulent or abusive behavior (Liou et al., 2008). They concluded that while all three methods were accurate, the classification tree model performs the best with an overall correct identification rate of 99% (Liou et al., 2008). Research by Yang and Hwang (2006) used supervised data mining approach to assess whether the providers followed defined clinical pathways. They assumed that deviations from clinical pathways could be an indication of fraudulent or abusive provision of care (Yang & Hwang, 2006).

Lin et al. (2008) applied unsupervised clustering methods on general physicians’ practice data of the National Health Insurance in Taiwan (Lin et al., 2008). They used ten indicators (features or attributes) to cluster physicians’ practice data. The indicators were amount of fee, number of cases, amount of prescription days, amount of visits per case, average consultation fee per case, average treatment fee per case, average drug fee per case, average fee per case, percentage of antibiotic prescriptions, and percentage of injection prescriptions. They identified and ranked critical clusters using expert opinions about the importance of clusters in affecting health expenditures. Finally, they illustrated managerial guidance based on expert opinions about the characteristics of each critical cluster (Lin et al., 2008). A Korean study aimed to identify abuse in 3705 internal medicine outpatient clinics’ claims (Shin, Park, Lee, & Jhee, 2012). This study gathered data from practitioner outpatient care claims submitted to a health insurance organization. They calculated a risk score for indicating the degree of likelihood of abuse by the providers; and then classified providers using a decision tree (Shin et al., 2012). As advantages, Shin et al used a simple definition of anomaly score and extracted 38 features for detecting abuse. They also provided a detailed explanation of the data mining process. Shan et al. (2009) used an outlier detection approach to assess optometrists’ claims to Medicare Australia based on methods introduced by Breunig et al. (2000) (Shan et al., 2009). They calculated one single measure, the Local Outlier Factor (LOF), indicating the degree of outlier-ness of each record. The complete definition and explanation of LOF can be found in the Breunig et al. (2000). They used the optometrists’ compliance history and feedback from experts to validate the findings (Shan et al., 2009). In another study, association rules mining were applied to examine claims of specialist physicians (Shan et al., 2008). The data was organized in transactions which were defined as all the items claimed or billed for one patient on one day by one specialist. Association rules are statements of the form if antecedent (s) then consequent (s). For example, if a physician prescribed drug A and drug B then he will prescribe drug C with a likelihood of 98%. They identified 215 association rules. They considered the specialists whose claims frequently broke the extracted rules as those with a higher risk of fraudulent behavior (Shan et al., 2008). The Australia’s Health Insurance Commission used an online-unsupervised learning algorithm (SmartSifter) to detect outliers in the utilization of pathology services in Medicare Australia (Yamanishi, Takeuchi, Williams, & Milne, 2004). Ekina et al. (2013) applied Bayesian co-clustering methods to identify potentially fraudulent providers and beneficiaries who might have perpetrated a “conspiracy fraud” (Ekina et al., 2013).

A study by Sokol, Garcia, Rodriguez, West, and Johnson (2001) explains the introductory steps of preparing and visualizing the data. These steps should be followed in any data mining approach. Usually these precursory steps need a large amount of work prior to the actual data mining. They used Health Care Financing Administration claims related to preventative services of mammography, bone density assessment and diabetic counseling (Sokol et al., 2001). Musal (2010) used Geo-location information and abnormally high utilization rates of services as indicators of fraudulent behavior.

### 5.5 Hybrid Supervised and Unsupervised Data Mining Methods

Hybrid methods of combining supervised and unsupervised methods also have been applied by some studies (Please see Table 1). Major and Riedinger (2002) tested an electronic fraud detection program that compared individual provider characteristics to their peers in identifying unusual provider behavior. Unsupervised learning is used to develop new rules and improve the identification process (Major & Riedinger, 2002). One study conducted a three step methodology for insurance fraud detection. They applied unsupervised clustering methods on insurance claims and developed a variety of (labeled) clusters. Then they used an algorithm based on a supervised classification tree and generated rules for the allocation of each record to clusters. They identified the most effective ’rules’ for future identification of abusive behaviors (Williams & Huang, 1997).

## 6. Conclusion

Our review demonstrates that the terms KDD and data mining are interpreted differently in different studies. These approaches contain an array of different methods and can be applied to different sets of problems (Maimon & Rokach, 2010). Development of practical guides may improve the uptake and usage of the methods and prevent errors and misuses of the techniques. Despite this limitation, the studies demonstrate that both supervised and unsupervised techniques have important merits in discovering different fraud strategies and schemes (Capelleveen, 2012).

Most of the identified literature focused more on the technical methods used in KDD and data mining, and paid little attention to the practical implications of their findings for health care managers and decision makers. An exception to this finding is the study by Lin et al. (2008) which provides a good example of a study that provides managerial implications of their findings for dealing with health care fraud. To improve the uptake of KDD and data mining methods, future studies should pay more attention to the policy implications of their findings.

It should be noted that fraud detection is only one part of a bigger program of combating health care fraud, abuse and waste (Rashidian et al., 2012). Fraud detection should note the pitfalls that health care delivery policies can create that might increase the possibility of fraud and abuse (Capelleveen, 2012). For example, fee for service payments can increase the quantity of delivered services (Chaix-Couturier, Durand-Zaleski, Jolly, & Durieux, 2000). This may act as a risk factor for abuse, and perhaps fraud in health care.

While fraud and abuse detection in health care is not merely an issue related to payments, most of the attention is towards frauds that result in unduly increasing costs and payments by the insurers. Further to this, any type of care not based on evidence is potentially susceptible for abuse or waste. We found one study that applied this logic to fraud detection (Yang & Hwang, 2006). More KDD research focusing on abuse resulting from non-evidence based provision of care is needed.

Interestingly, we found no studies that applied data mining methods on health care data for detecting insurer or payer fraud. Studies are needed to assess the potentials of these methods in detecting payer or insurer fraud.

We need more research on applying data mining methods in the context of low and middle-income countries. Many such countries have weak IT-based auditing systems, making data mining more difficult, and are probably more vulnerable to fraud and abuse. Where data is available, however, we think that low- and middle-income countries can use data mining techniques as an instrument for evaluating provider’s behavior. Applying unsupervised methods such as association rules induction and clustering are promising. These methods help compare each provider with peer-groups. For example, applying Apriori (rules induction) algorithms in prescription drugs of general physicians could result in a rule such as if a physician prescribed drug A and drug B then he will prescribe drug C with likelihood of 98%. This rule has originated from the behavior of all physicians. Hence, two per cent of physicians that break this rule should be investigated for the reasons behind this different behavior of prescription.

In conclusion, we recommend seven general steps to mining health care claims (or insurance claim) to detecting fraud and abuse (after preprocessing of data): 1). Identifying the most important attributes of data by expert domains (Sokol et al., 2001; Li et al., 2008) 2). Defining new features that are indicators of fraudulent or abusive behavior by expert domains or automated algorithms such as association rules induction (Li et al., 2008; Shan et al., 2008) 3). Identifying unusual records by outlier detection methods for detailed investigation (Shan et al., 2009) 4). Excluding outliers from the data and clustering (or re-clustering) records based on extracted features (Lin et al., 2008) 5). Identifying outlier cluster (s) and investigating records in those clusters in more detail and determining fraudulent or abusive records (e.g. by inspection) (Lin et al., 2008) 6). Designing supervised models based on labeled records of previous step and selecting the most discriminative features (Liou et al., 2008) 7). Applying supervised methods as a routine online processing task and applying unsupervised methods (outlier detection and clustering) in specific time periods for refining the previous steps and detecting new cases of fraud. Our recommended approach makes it possible to focus on a subset of claims instead of all claims, and is more likely to be useful in low resources setting where computerized data may have severe limitations.
